# Cross-sectional evaluation of leg-predominant relative segmental muscle mass differences in hospitalized older adults with sarcopenia

**DOI:** 10.3389/fnut.2026.1991967

**Published:** 2026-10-29

**Authors:** Weibin Yang, Bingjie Wu, Jie Li, Jiao Xue, Mengtian Guo, Xiaojuan Wang, Jing Chang

**Affiliations:** Department of Internal Medicine, Beijing Chao-yang Hospital, Capital Medical University, Beijing, China

**Keywords:** bioelectrical impedance analysis, hospitalized older adults, muscle mass distribution, sarcopenia, segmental muscle mass

## Abstract

**Background:**

Skeletal muscle mass index (SMI) summarizes total muscle mass but does not reflect regional distribution. This study investigated whether sarcopenia is associated with a preferential segmental pattern of lower muscle mass and whether regional fluid distribution contributes to this pattern.

**Methods:**

This cross-sectional study included 231 hospitalized adults aged ≥60 years, including 60 with sarcopenia. Segmental muscle mass and extracellular water-to-total body water ratio (ECW/TBW) were assessed using multifrequency bioelectrical impedance analysis. Sex-specific standardized segmental muscle mass scores were calculated using participants without sarcopenia as the reference. The primary analysis evaluated the interaction between sarcopenia status and body segment using generalized estimating equation models. Supporting analyses included within-group segmental comparisons and segment-specific regression models with adjustment for clinical covariates.

**Results:**

Among participants without sarcopenia, no consistent segmental distribution pattern was observed (Friedman *p* = 0.994). In contrast, participants with sarcopenia showed relatively lower standardized leg muscle mass compared with the arms and trunk (adjusted *p* = 0.037 and *p* < 0.001). After adjustment for clinical covariates, sarcopenia was associated with an additional relative reduction in leg muscle mass compared with the arm segment (*β* = −0.427; 95% CI, −0.708 to −0.147; *p* = 0.003). This association remained unchanged after further adjustment for segmental ECW/TBW (*β* = −0.425; 95% CI, −0.708 to −0.142; *p* = 0.003).

**Conclusion:**

In this hospitalized cohort, sarcopenia was associated with a greater relative muscle mass deficit in the legs than in the arms or trunk. This pattern was not substantially explained by regional fluid distribution. Segmental muscle mass assessment may provide additional information beyond conventional SMI, warranting prospective evaluation of its functional and prognostic relevance.

## Introduction

Sarcopenia is a common, age-related progressive skeletal muscle disorder associated with impaired mobility and increased risks of falls, fractures, and death (1, 2). Its core manifestations include decreased muscle strength and mass, often accompanied by impaired physical performance (3). As populations age, sarcopenia is becoming an increasing public health concern. Prevalence varies by population and diagnostic criteria, with a pooled estimate of 16.5% among community-dwelling older adults in Asia under the 2019 Asian Working Group for Sarcopenia (AWGS) criteria (4, 5). According to current consensus frameworks, low muscle mass is a prerequisite for confirming sarcopenia, and the skeletal muscle mass index (SMI) is the standard metric used in both clinical practice and research for its assessment (1, 2).

SMI provides a standardized measure of low appendicular muscle mass, but it does not show where muscle is relatively preserved or depleted (6). This limitation may be clinically relevant because a global measure can obscure regional differences in muscle involvement. Regional muscle groups may differ in their susceptibility to age-related muscle loss (7), and similar SMI values may therefore represent different segmental distributions of muscle mass. Although the field has broadened from diagnosing sarcopenia to preserving muscle health (1), evidence for BIA-based segmental muscle assessment remains relatively limited; for example, a recent scoping review of BIA-assessed trunk muscle mass identified only 7 eligible studies in older adults from 744 identified articles (8).

BIA provides segmental measurements at the bedside and therefore offers a practical approach to studying regional muscle distribution in hospitalized older adults (9), a setting where multidimensional vulnerability and geriatric syndromes are critically shaped by medication management and clinical status (10).

However, this convenience creates an important interpretive challenge. BIA estimates muscle mass from the electrical properties of tissue rather than measuring it directly, making the estimates sensitive to hydration and fluid distribution (11, 12). Moreover, segmental muscle mass and water indices are derived from the same impedance measurements. Regional differences in fluid distribution may therefore influence apparent differences in muscle mass and need to be considered when interpreting segmental comparisons (13).

Accordingly, this study examined whether sarcopenia is associated with a leg-predominant relative segmental pattern of lower muscle mass deficit rather than a uniform reduction across body regions, while also evaluating the potential contribution of segmental ECW/TBW. The central hypothesis was that sarcopenia would be associated with a disproportionate relative deficit in the legs, whereas no consistent segmental ordering would be observed without sarcopenia.

## Materials and methods

### Study design and participants

This single-center cross-sectional study consecutively enrolled inpatients aged 60 years or older admitted to the Department of Internal Medicine, Beijing Chao-yang Hospital, Capital Medical University, between July 2025 and March 2026. Exclusion criteria were: (1) diagnosed neuromuscular disease, such as myasthenia gravis or motor neuron disease; (2) marked edema, severe fluid retention, or other conditions likely to affect BIA measurements; (3) inability to complete the study assessments because of impaired consciousness, severe cognitive impairment, or other reasons; and (4) missing data required for sarcopenia classification or the primary segmental analyses, including the absence of both handgrip strength and physical-performance measurements.

### Sarcopenia definition

Sarcopenia status was classified according to the AWGS 2019 criteria (2). Low muscle mass corresponded to SMI of <7.0 kg/m^2^ in men and <5.7 kg/m^2^ in women; low muscle strength to handgrip strength of <28 kg in men and <18 kg in women; and impaired physical performance to a 6-m usual gait speed of <1.0 m/s or taking ≥12 s to complete the five-time chair stand test. A diagnosis of sarcopenia required low muscle mass together with low muscle strength and/or impaired physical performance. The AWGS low-muscle-mass criterion is based on total appendicular muscle mass and does not directly specify the relative distribution of muscle mass across body segments.

### Clinical data collection

Age, sex, height, and weight were recorded, and BMI was calculated as weight in kilograms divided by height in meters squared. Medical history was ascertained from medical records and patient interviews. Long-term medication use was abstracted from inpatient medication records; polypharmacy was defined as the regular use of five or more medications (14). Fasting venous blood was collected on the morning after admission for laboratory testing.

### Muscle strength and physical performance

Grip strength in the dominant hand was measured using a digital handgrip dynamometer (CAMRY EH101; Guangdong Senssun Weighing Apparatus Group Ltd., Zhongshan, China). Participants were seated with the elbow flexed at 90°and completed three maximal efforts; the highest value was retained (15). Usual gait speed was measured over 6 m on a flat, unobstructed indoor walkway with acceleration and deceleration zones. Two trials were completed, and the faster speed was retained; habitual walking aids were permitted (16). The five-time chair stand test was performed using a chair approximately 45 cm high. With their arms folded across the chest, participants completed five consecutive chair stands as quickly and safely as possible, and the total time was recorded (17). Physical performance tests were not fully integrated into the protocol at the outset of the study, and later, some patients still had missing data due to bedrest, medical contraindications, refusal, or inability to complete the tests safely.

### Body composition measurement

Body composition was assessed using a multifrequency bioelectrical impedance analyzer (InBody S10; InBody Co., Ltd., Seoul, Republic of Korea). The device uses direct segmental multifrequency bioelectrical impedance analysis to measure impedance at multiple frequencies in both arms, both legs, and the trunk. Segmental muscle mass and ECW/TBW were derived using the manufacturer’s algorithm. Trained investigators performed all measurements under standardized conditions. Before measurement, participants avoided strenuous activity, emptied their bladder, removed metal objects, and rested quietly before testing according to the manufacturer’s protocol. Multi-frequency BIA demonstrates excellent test–retest reliability for muscle mass and fluid parameters, with intraclass correlation coefficients ranging from 0.96 to 0.99 (18, 19). However, systematic differences between devices and algorithms, as well as the influence of abnormal fluid status, still warrant cautious interpretation (20, 21).

Arm muscle index (Arm MI; kg/m^2^) and leg muscle index (Leg MI; kg/m^2^) were calculated by dividing the summed muscle mass of both arms and both legs, respectively, by height squared. Trunk muscle index (Trunk MI; kg/m^2^) was calculated as trunk muscle mass divided by height squared. Arm and Leg ECW/TBW were calculated as the mean of the corresponding bilateral values, whereas Trunk ECW/TBW was taken directly from the device output.

To place anatomically distinct body segments on a comparable relative scale, segment-specific Z scores for MI and ECW/TBW were calculated using same-sex participants without sarcopenia as the internal reference. Z scores were calculated as follows: Z = (observed value −the corresponding segment-specific mean in the same-sex reference group)/the corresponding standard deviation. By design, the mean Z score for each segment was close to zero in the reference group. This standardization did not impose any within-participant ordering across the three segments.

### Sample size

All eligible patients were included, and no formal *a priori* sample size calculation was performed because this was an exploratory cross-sectional study. A post-hoc sensitivity analysis based on 10,000 simulations of the Friedman test (two-sided α = 0.05; three segments; *n* = 60 in the sarcopenia group) indicated that the study had approximately 80% power to detect a Kendall’s W of 0.09. The observed within-group effect size (W = 0.126) exceeded this threshold.

### Statistical analysis

All statistical analyses were performed using R version 4.5.3. Continuous variables are presented as medians (interquartile ranges) and categorical variables as counts (percentages). For group comparisons, continuous variables were analyzed using the Mann–Whitney U test, and categorical variables were analyzed using the Pearson χ^2^ test or Fisher’s exact test. Effect sizes (rank-biserial correlation or φ coefficient) and their 95% CIs were estimated using 2,000 bootstrap resamples.

Friedman tests compared arm, leg, and trunk MI Z scores separately in participants with and without sarcopenia. Kendall’s W was reported as the effect size, and pairwise comparisons used the Nemenyi *post hoc* test. Segmental differences were reported as Hodges–Lehmann paired location shifts with bootstrap 95% CIs. Arm and leg ECW/TBW Z scores were compared using the Wilcoxon signed-rank test. Because Z scores were standardized separately for each segment using same-sex participants without sarcopenia as the reference, the mean Z score for each segment in the reference group was centered at zero. This standardization did not impose any within-participant segmental ordering; the Friedman test was therefore used to assess whether a consistent ranking across segments was present.

Median quantile regression was used to examine the independent association between sarcopenia status and MI at each segment separately. Covariates were entered sequentially in two models. Model 1 included age, sex, and BMI. Model 2 additionally included serum albumin, chronic kidney disease (CKD), coronary artery disease (CAD), diabetes, and polypharmacy. All covariates were prespecified on the basis of prior evidence and clinical relevance. The regression coefficient represented the adjusted median difference in MI for participants with versus without sarcopenia. As a supplementary analysis independent of Z-score standardization, raw Leg MI/Arm MI and Leg MI/Trunk MI ratios were compared between groups and further evaluated using median quantile regression with the prespecified covariates. Rather than using propensity score matching, multivariable regression was used to directly adjust for confounding, thereby avoiding sample loss from matching and maintaining consistency with the adjustment framework of the subsequent generalized estimating equation (GEE) models.

Missing covariates were handled using multiple imputation by chained equations (MICE). Fifty imputed datasets were generated with 20 iterations each, using a random seed of 20,260,708. Continuous variables were imputed using predictive mean matching, and binary variables using logistic regression. The imputation model included the exposure, outcomes, prespecified covariates, and related BIA measures. Quantile regression models were fitted in each imputed dataset. Regression coefficients and variances were then pooled using Rubin’s rules. Koenker pseudo-R^2^ was used to describe the fit of the full model, reported as the median value across the 50 imputed datasets. The main models were also rerun in complete cases to assess robustness. As a separate BIA-derived measure, segmental ECW/TBW was not included in the quantile regression models.

GEE models examined the associations of sarcopenia status, body segment, and their interaction with segmental MI Z scores. Participants were treated as clusters. An exchangeable working correlation structure with robust standard errors was used. Participants without sarcopenia and the arm segment served as the reference categories. Model 1 included sarcopenia status, body segment, and their interaction. Model 2 additionally adjusted for the prespecified clinical covariates. Model 3 further included segmental ECW/TBW Z scores; given that MI and ECW/TBW are derived from the same impedance signal, Model 3 is defined *a priori* as a sensitivity analysis examining whether the sarcopenia-by-leg interaction is materially attenuated once between-segment hydration differences are accounted for, rather than as a conventional confounder adjustment. The sarcopenia-by-leg interaction was the primary term of interest. The overall interaction was assessed using a Wald test. As a sensitivity analysis, GEE models were repeated in participants aged ≥75 years using the original standardization and imputation procedures.

## Results

Of 248 patients who underwent body composition assessment, 17 lacked the muscle strength and physical performance data required to determine sarcopenia status and were excluded from the primary analysis. Included and excluded participants did not differ significantly in age, sex, BMI, SMI, or segmental MI (Supplementary Table S1). The final analytic sample comprised 231 participants: 171 without sarcopenia and 60 with sarcopenia (Figure 1). The median age was 71.0 years, and 118 participants (51.1%) were men. Compared with participants without sarcopenia, those with sarcopenia were older and had lower BMI and serum albumin levels. They also had higher prevalences of CKD, CAD, and polypharmacy. Arm, leg, and trunk MI were lower in the sarcopenia group, whereas ECW/TBW was higher in all three segments (*p* < 0.001; Table 1). No correction for multiple comparisons was applied to the baseline comparisons in Table 1, which are reported descriptively.

**Figure 1:**
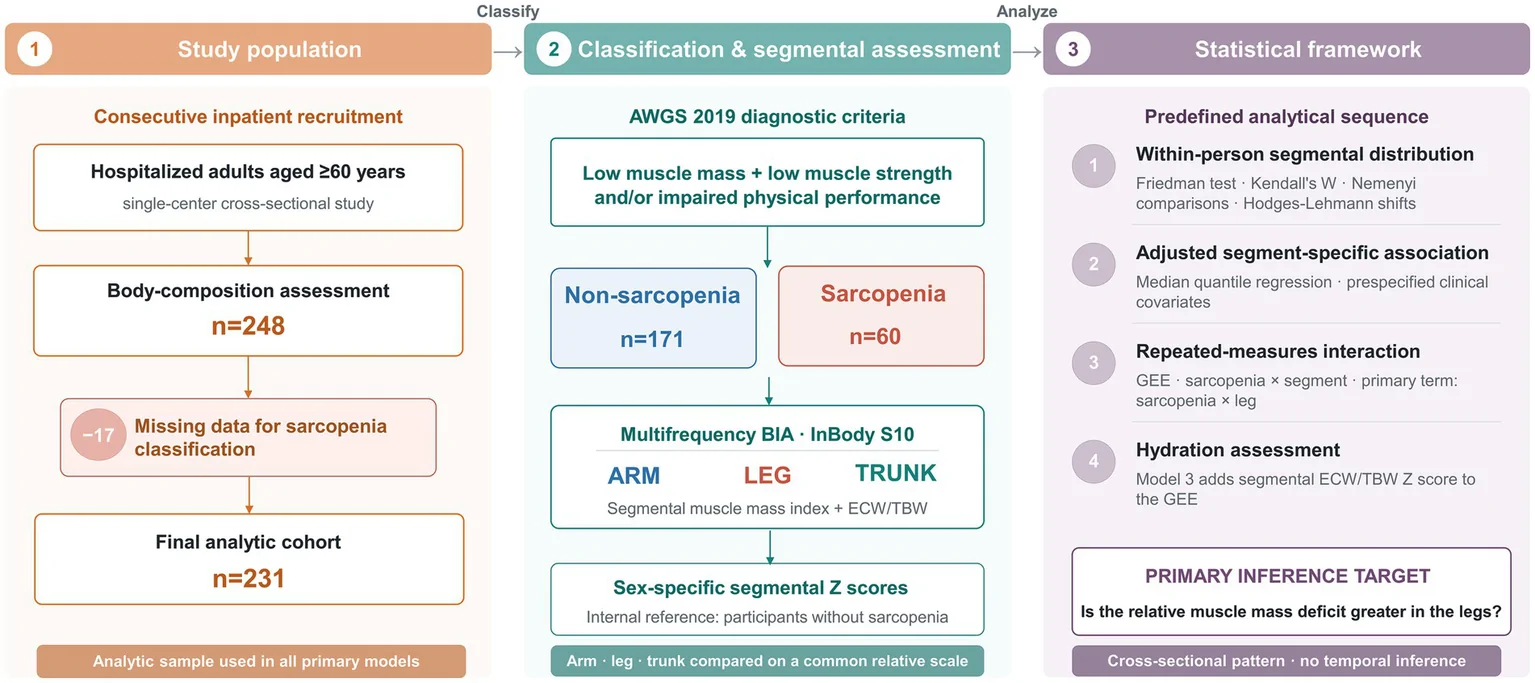
Study design and analytical framework. Of 248 patients who underwent body composition assessment, 17 were excluded because data required for sarcopenia classification were missing. The final analytic sample comprised 231 patients, including 171 without sarcopenia and 60 with sarcopenia. BIA, bioelectrical impedance analysis; ECW/TBW, extracellular water-to-total body water ratio; GEE, generalized estimating equations.

**Table 1:** Baseline characteristics and segmental BIA measures, by sarcopenia status.

Variable	Overall	Non-sarcopenia	Sarcopenia	Effect size (95% CI)	*p* value
A. Demographic and anthropometric characteristics					
Age, years	71.0 (65.0–81.0)	69.0 (63.0–76.0)	82.0 (72.5–88.5)	0.578 (0.439–0.696)	<0.001^*^
Sex, male	118 (51.1%)	89 (52.0%)	29 (48.3%)	−0.033 (−0.154–0.101)	0.621^†^
BMI, kg/m^2^	23.4 (21.5–25.4)	24.1 (22.5–26.4)	20.9 (18.6–22.9)	−0.603 (−0.722 to −0.463)	<0.001^*^
B. Nutritional and clinical characteristics					
Albumin, g/L (*n* = 223)	40.4 (38.0–43.5)	41.0 (38.8–43.9)	38.3 (35.6–41.1)	−0.372 (−0.530 to −0.195)	<0.001^*^
CKD (*n* = 226)	28 (12.4%)	14 (8.4%)	14 (23.3%)	0.200 (0.056–0.343)	0.003^†^
Diabetes (*n* = 226)	76 (33.6%)	54 (32.5%)	22 (36.7%)	0.039 (−0.088–0.168)	0.561^†^
Hypertension (*n* = 226)	139 (61.5%)	99 (59.6%)	40 (66.7%)	0.064 (−0.059–0.185)	0.338^†^
Coronary artery disease (*n* = 226)	71 (31.4%)	46 (27.7%)	25 (41.7%)	0.133 (−0.002–0.266)	0.046^†^
Polypharmacy (*n* = 225)	77 (34.2%)	50 (30.3%)	27 (45.0%)	0.137 (0.002–0.279)	0.040^†^
C. Sarcopenia-defining components					
SMI, kg/m^2^	6.82 (6.16–7.72)	7.21 (6.37–7.96)	5.65 (5.28–6.65)	−0.692 (−0.794 to −0.574)	<0.001^*^
Handgrip strength, kg (*n* = 214)	21.0 (15.9–29.4)	24.2 (18.2–32.1)	15.6 (10.9–21.4)	−0.577 (−0.697 to −0.454)	<0.001^*^
Gait speed, m/s (*n* = 115)	0.98 (0.82–1.10)	1.01 (0.87–1.12)	0.85 (0.63–0.98)	−0.485 (−0.681 to −0.269)	<0.001^*^
5-time chair stand test, s (*n* = 89)	11.9 (10.6–13.5)	11.6 (10.1–13.3)	13.8 (11.7–15.1)	0.363 (0.089–0.623)	0.032^*^
D. Segmental BIA phenotype					
Arm MI, kg/m^2^	1.71 (1.52–2.01)	1.82 (1.58–2.13)	1.45 (1.23–1.70)	−0.610 (−0.730 to −0.482)	<0.001^*^
Leg MI, kg/m^2^	5.08 (4.59–5.70)	5.35 (4.81–5.84)	4.21 (3.98–4.93)	−0.704 (−0.800 to −0.587)	<0.001^*^
Trunk MI, kg/m^2^	7.42 (6.90–8.16)	7.67 (7.09–8.45)	6.67 (5.96–7.29)	−0.657 (−0.768 to −0.538)	<0.001^*^
Arm ECW/TBW	0.381 (0.377–0.385)	0.380 (0.376–0.384)	0.383 (0.380–0.390)	0.357 (0.191–0.516)	<0.001^*^
Leg ECW/TBW	0.394 (0.385–0.404)	0.390 (0.384–0.400)	0.405 (0.394–0.415)	0.461 (0.306–0.611)	<0.001^*^
Trunk ECW/TBW	0.390 (0.382–0.398)	0.387 (0.381–0.394)	0.399 (0.390–0.407)	0.506 (0.348–0.651)	<0.001^*^

Data are median (IQR) or *n* (%). Sample sizes for grip strength, gait speed, and chair stand test: 156/58, 95/20, and 75/14 in non-sarcopenia/sarcopenia groups. Group comparisons: Mann–Whitney U for continuous; Pearson χ^2^ or Fisher’s exact for categorical. Effect sizes (rank-biserial for continuous; φ for binary) with 95% CIs from 2,000 bootstrap resamples. Binary variables coded as 1 for sarcopenia, male sex, or condition; positive φ = higher prevalence in sarcopenia group. *Mann–Whitney U; ^†^Pearson χ^2^.

Participants without sarcopenia showed no consistent segmental ordering (Friedman *p* = 0.994; Kendall’s W < 0.001). Among participants with sarcopenia, median MI Z scores were −1.64 for the arms, −2.09 for the legs, and −1.60 for the trunk, with a significant overall difference across segments (Friedman *p* < 0.001; Kendall’s W = 0.126). *Post hoc* comparisons showed that leg MI Z scores were lower than arm MI Z scores (Hodges–Lehmann paired difference, −0.45; adjusted *p* = 0.037) and trunk MI Z scores (paired difference, −0.46; adjusted *p* < 0.001). Arm and trunk MI Z scores did not differ significantly (*p* = 0.357). Within the sarcopenia group, leg ECW/TBW Z scores were also higher than arm ECW/TBW Z scores (Hodges–Lehmann paired difference, 0.31; *p* = 0.016). No corresponding difference was observed among participants without sarcopenia (paired difference, 0.01; *p* = 0.866) (Table 2; Figure 2).

**Table 2:** Within-group segmental Z-score comparisons.

Measure	Non-sarcopenia (*n* = 171)	Sarcopenia (*n* = 60)
A. Segmental MI Z-scores		
Arm MI Z-score median (IQR)	−0.09 (−0.64–0.54)	−1.64 (−2.49 to −1.15)
Leg MI Z-score median (IQR)	−0.13 (−0.77–0.57)	−2.09 (−2.59 to −1.74)
Trunk MI Z-score median (IQR)	−0.11 (−0.57–0.50)	−1.60 (−2.54 to −1.02)
Friedman χ^2^(df = 2)	0.012	15.100
Kendall’s W	<0.001	0.126
Overall *p*	0.994	<0.001
Leg−Arm Hodges-Lehmann paired difference (bootstrap 95% CI)	0.01 (−0.10–0.13)	−0.45 (−0.71 to −0.18)
Leg−Arm adjusted *p*	0.998	0.037
Leg−Trunk Hodges-Lehmann paired difference (bootstrap 95% CI)	0.01 (−0.11–0.14)	−0.46 (−0.72 to −0.18)
Leg−Trunk adjusted *p*	0.998	<0.001
Arm−Trunk Hodges-Lehmann paired difference (bootstrap 95% CI)	0.00 (−0.02–0.02)	−0.03 (−0.06–0.02)
Arm−Trunk adjusted *p*	0.994	0.357
B. Paired comparison of leg and arm ECW/TBW Z-scores		
paired *n*	171	60
Arm ECW/TBW Z-score median (IQR)	−0.05 (−0.64–0.55)	0.55 (−0.01–1.60)
Leg ECW/TBW Z-score median (IQR)	−0.08 (−0.72–0.61)	0.85 (0.17–1.69)
Leg−Arm Hodges-Lehmann paired difference (bootstrap 95% CI)	0.01 (−0.14–0.16)	0.31 (0.06–0.56)
Paired-test *p* value	0.866	0.016

Data are median (IQR). Z scores were standardized using same-sex non-sarcopenic participants as the reference for each segment. This standardization centers Z scores in the non-sarcopenia group near zero but does not restrict the ranking of segments within an individual. In Panel A, overall differences were assessed by Friedman test with Kendall’s W; pairwise comparisons used Nemenyi test. Hodges–Lehmann paired differences and 95% CIs from 2,000 bootstrap samples. In Panel B, leg–arm ECW/TBW Z scores were compared by paired Wilcoxon test. Lower MI Z = lower relative muscle mass; higher ECW/TBW Z = greater relative fluid elevation. MI, muscle index; ECW/TBW, extracellular water to total body water ratio.

**Figure 2:**
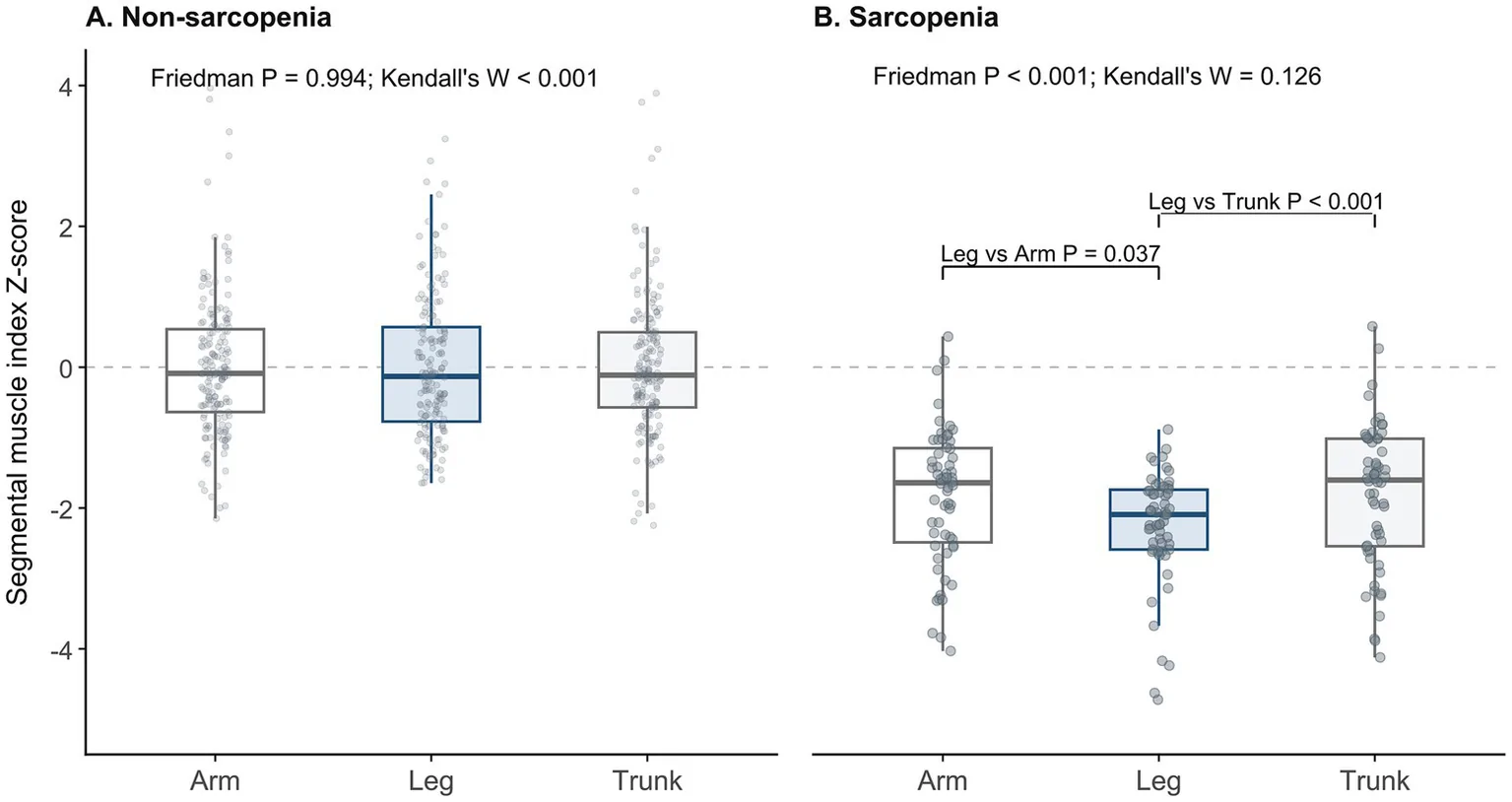
Segmental MI *Z*-score distributions, by sarcopenia status. **(A)** Non-sarcopenia group (*n* = 171); **(B)** Sarcopenia group (*n* = 60). Box plots show medians and *IQRs*; whiskers extend to 1.5 x *IQR*; points are individual values. Horizontal dashed line at *Z* = 0. Overall differences across segments were assessed by Friedman test, with pairwise comparisons by Nemenyi test. Lower MI *Z*-scores indicate lower muscle mass relative to the same-sex non-sarcopenic internal reference. MI, muscle index.

Median regression showed that sarcopenia was associated with lower muscle mass across all three segments (Table 3). In the age-, sex-, and BMI-adjusted model (Model 1), the largest absolute difference was observed for the leg (*β* = −0.638; 95% CI, −0.756 to −0.519; *p* < 0.001), and this association remained materially unchanged after further adjustment in Model 2 (*β* = −0.603; 95% CI, −0.717 to −0.490; *p* < 0.001). Unadjusted Leg MI/Arm MI was higher, whereas Leg MI/Trunk MI was lower in participants with sarcopenia (Supplementary Table S2). After multivariable adjustment, both ratios were lower in the sarcopenia group (Supplementary Table S3).

**Table 3:** Quantile regression models for segment-specific MI.

Segment-specific muscle index	Model 1				Model 2			
	*n*	*β* (95% CI)	*p*	Pseudo-R^2^	*n*	*β* (95% CI)	*p*	Pseudo-R^2^
Arm MI	231	−0.100 (−0.141 to −0.059)	<0.001	0.678	231	−0.120 (−0.152 to −0.087)	<0.001	0.685
Leg MI	231	−0.638 (−0.756 to −0.519)	<0.001	0.584	231	−0.603 (−0.717 to −0.490)	<0.001	0.590
Trunk MI	231	−0.250 (−0.353 to −0.146)	<0.001	0.688	231	−0.282 (−0.380 to −0.184)	<0.001	0.694

Quantile regression at τ = 0.50. The *β* coefficients are adjusted median differences in MI for sarcopenia vs. non-sarcopenia. Model 1 adjusted for age, sex, and BMI; Model 2 additionally adjusted for albumin, CKD, CAD, diabetes, and polypharmacy. Missing covariates imputed by MICE (50 datasets); estimates pooled by Rubin’s rules. Koenker pseudo-R^2^ reported as median across imputed datasets. Segmental ECW/TBW not included (Table 4, Model 3).

GEE analysis showed a significant interaction between sarcopenia status and body segment (overall interaction *p* = 0.011; Table 4). In the covariate-adjusted model, participants with sarcopenia showed an additional reduction in leg MI Z score relative to arm MI Z score compared with those without sarcopenia (*β* = −0.427; 95% CI, −0.708 to −0.147; *p* = 0.003), whereas trunk MI did not differ significantly from arm MI. The overall interaction remained significant (*p* = 0.013). The principal segmental findings are summarized in Figure 3. The sarcopenia-by-leg interaction remained significant in participants aged ≥75 years (*n* = 90; *β* = −0.671, 95% CI: −1.056 to −0.285; *p* < 0.001; Supplementary Table S4).

**Table 4:** Generalized estimating equation models for segmental MI Z-scores.

Predictor	Model 1			Model 2			Model 3		
	*β* (95% CI)	SE	*p*	*β* (95% CI)	SE	*p*	*β* (95% CI)	SE	*p*
Sarcopenia	−1.828 (−2.111 to −1.544)	0.145	<0.001	−0.827 (−1.060 to −0.593)	0.119	<0.001	−0.827 (−1.060 to −0.594)	0.119	<0.001
**Sarcopenia × Leg**	**−0.427 (−0.708 to −0.147)**	**0.143**	**0.003**	**−0.427 (−0.708 to −0.147)**	**0.143**	**0.003**	**−0.425 (−0.708 to −0.142)**	**0.144**	**0.003**
Sarcopenia × Trunk	0.012 (−0.035–0.060)	0.024	0.614	0.012 (−0.035–0.060)	0.024	0.614	0.016 (−0.032–0.063)	0.024	0.521
Age				−0.028 (−0.037 to −0.018)	0.005	<0.001	−0.027 (−0.036 to −0.018)	0.005	<0.001
Male sex				−0.250 (−0.395 to −0.105)	0.074	<0.001	−0.250 (−0.395 to −0.105)	0.074	<0.001
BMI				0.203 (0.174–0.233)	0.015	<0.001	0.203 (0.174–0.233)	0.015	<0.001
Albumin				−0.007 (−0.031–0.016)	0.012	0.525	−0.008 (−0.031–0.015)	0.012	0.505
CKD				−0.204 (−0.459–0.050)	0.130	0.116	−0.202 (−0.458–0.053)	0.131	0.121
Coronary artery disease				0.125 (−0.047–0.298)	0.088	0.155	0.126 (−0.045–0.297)	0.087	0.148
Diabetes				−0.133 (−0.274–0.007)	0.072	0.063	−0.133 (−0.273–0.008)	0.072	0.065
Polypharmacy				0.055 (−0.111–0.222)	0.085	0.514	0.056 (−0.111–0.223)	0.085	0.510
Segmental ECW/TBW Z-score							−0.009 (−0.049–0.032)	0.021	0.679

GEE with exchangeable working correlation and robust standard errors for repeated arm, leg, and trunk MI Z-score measurements within participants. Reference: arm segment and non-sarcopenia group. Model 1: sarcopenia status, segment, and their interaction. Model 2: additionally adjusted for age, sex, BMI, albumin, CKD, CAD, diabetes, and polypharmacy. Model 3: sensitivity analysis additionally adjusted for segmental ECW/TBW Z-score (defined a priori as a sensitivity model, not a primary confounder adjustment, because MI and ECW/TBW share the same impedance signal). Results from 50 multiply imputed datasets pooled by Rubin’s rules. Sarcopenia × Leg coefficient attenuated by 0.5% from Model 2 to Model 3. Bold values indicate the sarcopenia × leg interaction, the prespecified primary term of interest.

**Figure 3:**
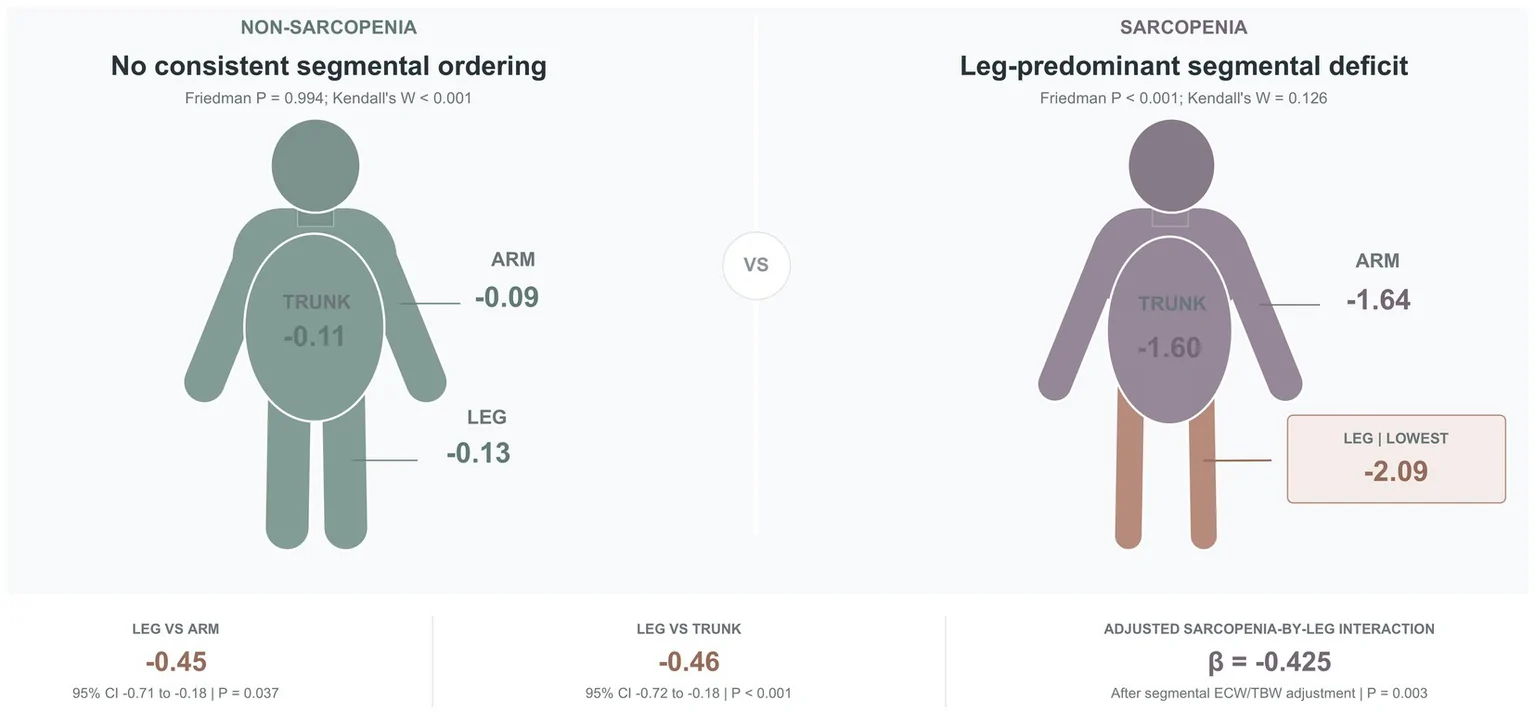
Leg-predominant relative segmental muscle mass differences by sarcopenia status.

## Discussion

Across three complementary analytical approaches, sarcopenia was associated with lower segmental muscle mass in the arms, legs, and trunk, but the relative deficit was greatest in the legs. The absence of a reproducible segmental ranking among participants without sarcopenia, together with the sarcopenia-by-leg interaction and its minimal attenuation after inclusion of segmental ECW/TBW, supports a leg-predominant relative segmental muscle mass deficit within this hospitalized cohort. This finding should be interpreted as a cross-sectional distributional pattern, rather than evidence that leg muscle loss begins earlier or progresses faster than muscle loss in other segments.

Previous studies provide some support for this nonuniform pattern. In a study of 1,370 community-dwelling Japanese adults aged 65 to 90 years, Asano et al. found age-related declines in muscle mass, strength, physical performance, and muscle quality. However, the patterns varied by sex and muscle region (22). Further MRI data from 50,332 UK Biobank participants aged 45 to 84 years showed a clear age gradient, with lower thigh fat-free muscle volume and greater intramuscular fat at older ages, suggesting that leg muscle aging involves not only loss of mass but also alterations in tissue composition (23). Another study of community-dwelling older adults found that regional muscle mass and muscle quality measures of the quadriceps and triceps surae were associated with different physical performance tests. By contrast, overall SMI was not associated with any performance measure in multivariable models (24). Together, these studies indicate that overall and segmental muscle measures should not be assumed to convey the same biological or functional information. The present findings extend this evidence by showing that relative segmental muscle distribution also differs according to sarcopenia status.

Because low SMI is part of the definition of sarcopenia, an overall reduction in appendicular muscle mass is expected in the sarcopenia group. The more informative observation is the altered within-person distribution: the relative muscle mass deficit was greater in the legs than in the arms and trunk in sarcopenia, whereas no stable segmental ordering was present in the comparison group. The low-muscle-mass criterion concerns summed appendicular muscle mass, whereas the primary contrast (sarcopenia × leg interaction) compares within-person leg–arm differences in standardized MI Z-scores between groups. Although the diagnostic criterion does not prescribe a particular segmental pattern, shared BIA-derived measurements may introduce mathematical coupling. This pattern is consistent with imaging evidence that age-related change is muscle-group specific (25, 26) and BIA studies showing regional differences across sarcopenia phenotypes (27, 28). The interaction-based analysis therefore adds a distributional contrast beyond the expected overall reduction in appendicular muscle mass. This distinction may be clinically relevant because leg muscle is central to mobility and functional independence.

Several mechanisms provide biological plausibility for a leg-predominant relative pattern. Leg antigravity muscles, many of which contain a substantial proportion of type II fibers, are vulnerable to disuse, denervation, and age-related remodeling (29, 30). Human multiomics and biopsy studies have identified selective loss of type II fiber-associated myonuclei, reduced regenerative capacity, and increasingly inflammatory, fibrotic, and morphologically irregular muscle with aging (31, 32). Sarcopenia is also associated with leg motor-unit loss, reduced motoneuron excitability, and altered recruitment during functional tasks (33–35). In parallel, reduced standing, walking, and transfer activity diminishes mechanical loading of the legs (36). During hospitalization, acute illness and bed rest may further reduce habitual weight bearing, potentially amplifying disuse-related changes in the legs. Together, these pathways provide biological plausibility for greater vulnerability of leg muscle, although the underlying mechanisms require further investigation.

Regional fluid distribution was an important alternative explanation because BIA estimates muscle mass from tissue electrical properties and because segmental muscle mass and ECW/TBW are derived from the same impedance signal. Higher ECW/TBW has been associated with sarcopenia and frailty (37, 38), and fluid correction can materially alter BIA-derived appendicular muscle estimates in patients with fluid retention (39). In the present cohort, leg ECW/TBW was relatively higher than arm ECW/TBW among participants with sarcopenia; however, adding segmental ECW/TBW Z score changed the sarcopenia-by-leg estimate by only 0.5%. Thus, measured regional fluid distribution did not materially attenuate the observed interaction within this BIA framework. Although residual hydration effects cannot be completely excluded, the stability of the interaction after ECW/TBW adjustment supports the robustness of the observed segmental pattern.

Several design features strengthen the interpretation of these findings. Sex-specific internal standardization and complementary nonparametric and repeated-measures analyses were applied. Within-group patterns were distinguished from between-group interactions, and segmental ECW/TBW was directly evaluated as a potential alternative explanation. The main contribution of this study is the regional information it adds beyond SMI. Whereas SMI identifies whether appendicular muscle mass is low overall, segmental assessment may additionally indicate where the relative muscle mass deficit is greatest. The leg-predominant pattern may be clinically relevant because leg muscle plays a central role in transfers, standing, walking, and independent mobility. Recent evidence in hospitalized older cohorts further underscores that evaluating sarcopenia and muscle status through objective measures is vital for understanding clinical outcomes such as functional impact and geriatric complications (40, 41). These findings provide a rationale for longitudinal studies examining whether a greater relative leg deficit is associated with mobility decline, subsequent need for walking aids or wheelchair use, and broader functional dependence. Such outcomes should also be considered alongside nutritional status and body composition, given their reported links to sarcopenia (42) and daily functioning (43). Studies integrating segmental muscle assessment with nutritional status, leg strength, physical performance, imaging, and rehabilitation outcomes may further clarify its clinical and functional significance and determine whether segmental assessment provides useful information beyond conventional SMI.

The study has several limitations. First, the cross-sectional design precludes assessment of temporal changes or causal relationships. Second, this single-center cohort consisted exclusively of older patients admitted to an internal medicine ward, which may limit generalizability of the findings to healthy or community-dwelling older adults. Third, reference imaging such as CT or MRI was unavailable; therefore, segmental muscle mass estimates derived from the InBody S10 could not be compared with direct anatomical measurements. Shared BIA-derived measures may introduce mathematical coupling. Fourth, physical performance data were incomplete due to limited early testing, bed rest, refusal, or medical contraindications, restricting assessment of the functional relevance of the observed segmental pattern.

## Conclusion

In hospitalized older adults, sarcopenia was associated with a greater relative leg muscle mass deficit than in the arms or trunk, whereas no consistent segmental pattern was observed without sarcopenia. This pattern persisted after adjustment for clinical covariates and was minimally affected by segmental ECW/TBW. Segmental muscle mass assessment may provide additional information beyond conventional SMI, although its clinical and functional significance requires confirmation in prospective studies with longitudinal follow-up.

## Data Availability

The original contributions presented in the study are included in the article/Supplementary material, further inquiries can be directed to the corresponding authors.
