# Supplementary material for: Cross-sectional evaluation of leg-predominant relative segmental muscle mass differences in hospitalized older adults with sarcopenia
Source: Front Nutr. 2026 Oct 29;13:1991967. doi: 10.3389/fnut.2026.1991967 (PMC13754836; doi:10.3389/fnut.2026.1991967)
Supplement: Supplementary file 1 [file Supplementary_file_1.pdf]

Supplementary Material

Supplementary Table S1. Characteristics of included and excluded participants

Variable	Overall (n = 248)	Included (n = 231)	Excluded (n = 17)	Effect size (95% CI)	P value
Age, years	71.5 (65.0–80.5)	71.0 (65.0–81.0)	76.0 (65.0–79.0)	0.014 (−0.272–0.280)	0.925*
Sex, male	127 (51.2%)	118 (51.1%)	9 (52.9%)	0.009 (−0.124–0.131)	0.882†
BMI, kg/m²	23.4 (21.5–25.7)	23.4 (21.5–25.4)	25.5 (22.2–27.3)	0.270 (−0.036–0.557)	0.064*
SMI, kg/m²	6.83 (6.17–7.73)	6.82 (6.16–7.72)	7.05 (6.50–7.93)	0.108 (−0.138–0.353)	0.457*
Arm MI, kg/m²	1.71 (1.52–2.02)	1.71 (1.52–2.01)	1.89 (1.70–2.13)	0.224 (−0.025–0.458)	0.123*
Leg MI, kg/m²	5.08 (4.59–5.70)	5.08 (4.59–5.70)	5.14 (4.58–5.69)	0.033 (−0.215–0.264)	0.819*
Trunk MI, kg/m²	7.43 (6.92–8.19)	7.42 (6.90–8.16)	7.75 (7.37–8.65)	0.232 (−0.033–0.483)	0.111*

Data are median (IQR) or n (%). Group comparisons: * Mann–Whitney U test; † Pearson χ^2 test. Effect sizes are rank-biserial correlations for continuous variables and φ coefficients for sex, with 95% CIs from 2,000 bootstrap resamples. BMI, body mass index; SMI, skeletal muscle mass index; MI, muscle index.

Supplementary Table S2. Raw segmental MI ratios by sarcopenia status

Variable	Overall	Non-sarcopenia	Sarcopenia	Effect size (95% CI)	P value
Leg MI/Arm MI	2.929 (2.727–3.150)	2.926 (2.698–3.083)	3.006 (2.819–3.375)	0.246 (0.059–0.421)	0.005*
Leg MI/Trunk MI	0.682 (0.651–0.713)	0.687 (0.656–0.715)	0.660 (0.623–0.706)	−0.203 (−0.388–−0.005)	0.019*

Data are median (IQR); n=171/60 in the non-sarcopenia/sarcopenia groups. Ratios were calculated from raw MI values. Effect sizes are rank-biserial correlations with 95% CIs from 2,000 bootstrap resamples. *Mann–Whitney U test. MI, muscle index.

Supplementary Table S3. Covariate-adjusted models for raw segmental MI ratios

	Model 1				Model 2			
**Segmental MI ratio**	**n**	**β (95% CI)**	**P**	**Pseudo-R^2**	**n**	**β (95% CI)**	**P**	**Pseudo-R^2**
Leg MI/ Arm MI	231	−0.137 (−0.243–−0.031)	0.011	0.307	231	−0.141 (−0.246–−0.036)	0.009	0.334
Leg MI/ Trunk MI	231	−0.055 (−0.079–−0.031)	<0.001	0.149	231	−0.060 (−0.078–−0.041)	<0.001	0.178

Median quantile regression at τ=0.50. β represents the adjusted median difference in MI ratios for sarcopenia versus non-sarcopenia. Model 1 adjusted for age, sex, and BMI; Model 2 additionally adjusted for albumin, CKD, CAD, diabetes, and polypharmacy. Model 2 estimates were pooled across 50 imputed datasets using Rubin's rules; Koenker pseudo-R^2 was reported as the median across datasets. MI, muscle index; BMI, body mass index; CKD, chronic kidney disease; CAD, coronary artery disease.

Supplementary Table S4. GEE interaction analyses in participants aged ≥75 years

	Model 1			Model 2			Model 3		
**Predictor**	**β (95% CI)**	**SE**	**P**	**β (95% CI)**	**SE**	**P**	**β (95% CI)**	**SE**	**P**
Sarcopenia	−1.606 (−2.028–−1.185)	0.215	<0.001	−0.803 (−1.134–−0.473)	0.169	<0.001	−0.798 (−1.127–−0.469)	0.168	<0.001
**Sarcopenia × Leg**	**−0.671 (−1.056–−0.285)**	**0.197**	**<0.001**	**−0.671 (−1.056–−0.285)**	**0.197**	**<0.001**	**−0.673 (−1.057–−0.288)**	**0.196**	**<0.001**
Sarcopenia × Trunk	0.041 (−0.024–0.106)	0.033	0.214	0.041 (−0.024–0.106)	0.033	0.214	0.047 (−0.019–0.113)	0.034	0.166
Age				−0.030 (−0.050–−0.009)	0.010	0.004	−0.028 (−0.049–−0.008)	0.010	0.006
Male sex				−0.371 (−0.596–−0.146)	0.115	0.001	−0.367 (−0.590–−0.144)	0.114	0.001
BMI				0.190 (0.149–0.231)	0.021	<0.001	0.191 (0.150–0.232)	0.021	<0.001
Albumin				−0.007 (−0.045–0.031)	0.019	0.714	−0.010 (−0.048–0.027)	0.019	0.596
CKD				−0.400 (−0.735–−0.065)	0.171	0.019	−0.391 (−0.730–−0.053)	0.173	0.023
Coronary artery disease				0.068 (−0.201–0.336)	0.137	0.622	0.077 (−0.185–0.340)	0.134	0.563
Diabetes				0.038 (−0.185–0.261)	0.114	0.741	0.039 (−0.181–0.259)	0.112	0.729
Polypharmacy				0.153 (−0.104–0.409)	0.131	0.244	0.165 (−0.089–0.418)	0.129	0.203
Segmental ECW/TBW Z-score							−0.039 (−0.094–0.017)	0.029	0.176

The subgroup comprised 90 participants (49 non-sarcopenia and 41 sarcopenia). Original full-cohort Z-score references and 50 imputed datasets were retained. GEE used an exchangeable working correlation and robust standard errors for repeated segmental MI Z-scores, with arm and non-sarcopenia as references. Model 1: sarcopenia, segment, and their interaction. Model 2: additionally adjusted for age, sex, BMI, albumin, CKD, CAD, diabetes, and polypharmacy.

Model 3: additionally adjusted for segmental ECW/TBW Z-score as a sensitivity model, because MI and ECW/TBW share the same impedance signal. Estimates were pooled using Rubin's rules. Overall interaction P = 0.003 in all models.
